# Awareness and Reporting of Sharps Injuries: A Study Involving Dental Students, Trainees, and Assistants in a Dental Teaching Hospital in Saudi Arabia

**DOI:** 10.7759/cureus.52843

**Published:** 2024-01-24

**Authors:** Ehab Azab, Ibtesam K Afifi

**Affiliations:** 1 Basic and Clinical Oral Sciences, Faculty of Dental Medicine, Umm Al-Qura University, Makkah, SAU; 2 Medical Microbiology & Immunology, Faculty of Medicine, Tanta University, Tanta, EGY

**Keywords:** trainees, occupational hazards, proper knowledge, exposure, needle stick

## Abstract

Background: Sharps injury constitutes one of the major occupational hazards in dental practice with practitioners under training being the most exposed group. This study aimed to assess the level of awareness about sharps injury, its prevalence, and reporting rates among dental students, trainees, and assistants.

Methods: The study was conducted at the Dental Teaching Hospital, Umm Al Qura University, Saudi Arabia, through an online self-designed questionnaire which comprised 21 items. Data was collected, tabulated, and statistically analyzed.

Results: Among 182 responding participants, the mean awareness score was satisfactory in 117 participants (64.3%) and average in 64 (35.2%). Exposure to sharps injury in the last 24 months was reported by 31.3% (n=20) with needle stick injury being the most frequent cause and only 59.6% (n=34) reported the injury. Interns and sixth-year students were the most injured participants. There was a significant difference in knowledge between exposed and non-exposed participants regarding the safe technique of recapping needles (p=0.037). After the injury, 77.2% (n=44) of participants washed their hands with soap and water.

Conclusions: A considerable percentage of study participants have average to satisfactory awareness about the risk of sharps injury with a high under-reporting rate. So, comprehensive preclinical education and training must be provided to our hospital’s students, trainees, and assistants to increase awareness about potentially risky behavior. More orientation about reporting and its role in prevention is highly recommended to ensure safe practice and improve the quality of dental care.

## Introduction

A sharps injury is defined as an unintentional penetrating wound of the skin by sharp items such as hollow-bore needles, scalpels, lancets, razor blades, scissors, or metal wire [1]. It constitutes one of the most serious occupational hazards in dental practice and the major contributing factor to acquiring infectious diseases. It could accidentally happen in different circumstances during treating patients or during needle disposal [2].

In their daily work, dental students, trainees, and dental assistants are frequently exposed to sharps injuries or needle stick injuries (NSI) by needles contaminated with blood and saliva while managing their patients under restricted visibility. Thus, there is a potential risk of infection by blood-borne pathogens, mostly hepatitis B virus (HBV), hepatitis C virus (HCV), and HIV [3,4]. The most important contributing factors to NSI include the routine use of anesthesia needles, surgical hypodermic needles, and suture needles as well as the needle design, bad recapping practice, or failure to dispose of needles in the safety box [5].

The World Health Organization (WHO) previously reported that approximately three out of 35 million healthcare workers (HCWs) worldwide get sharps injuries per year and are thus exposed to blood-borne pathogens [6]. It was estimated to be the highest among dental practitioners, compared with other HCWs [7]. A WHO report on annual occupational exposures to sharps injuries revealed that more than two million injuries occurred among 300 dental training students [8]. Predisposing factors that could lead to such injuries during dental practice are insufficient training, lack of experience, overloaded tasks, and fatigue [3]. Due to the fact that dental students, trainees, and dental assistants are the categories having the lowest experience among dental practitioners, thus, knowledge and awareness about sharps injuries are particularly important among them to avoid transmission of bloodborne diseases [9]. WHO statistics showed that these injuries among dental training students cause HCV, HBV, and HIV at a rate of 16,000, 66,000, and 1,000 cases per year, respectively [7].

Dental students and trainees are the groups most vulnerable to sharps injury due to the nature of dental training in different departments during the clinical sessions to complete their clinical requirements. Additionally, dental assistants are also among the higher-risk groups as they are delegated to provide chair-side support during the various steps of dental treatment in addition to preparing instruments and equipment for dental procedures and maintaining clinics’ environments [10].

Only a few previous studies have been conducted in Saudi Arabia to assess knowledge and experience on NSI/sharps injury among dental practitioners. However, one of these studies targeted senior students in both dentistry and pharmacy colleges in Riyadh [11]. The second study was conducted on dental assistants recruited from randomly selected private clinics in Jeddah, Saudi Arabia [12]. A third study was conducted only on undergraduate dental students and interns at Qassim University from December 2015 to February 2016 [13].

To the best of our knowledge, no published study in Saudi Arabia involved all clinical training dental students, interns, trainees, and dental assistants at a university hospital or assessed their reporting rates of sharps injuries. Thus, this study was conducted to determine the level of awareness and knowledge about occupational sharps injury risk and practices related to it as well as to assess its prevalence and reporting rates in the previous 24 months (from March 1, 2021, to March 1, 2023) among all clinical year dental students, interns, trainees, and dental assistants in Dental Teaching Hospital, Umm Al-Qura University, Makkah, Saudi Arabia. 

## Materials and methods

Study design and population

This cross-sectional study was conducted in the Dental Teaching Hospital, Umm Al-Qura University, Makkah, Saudi Arabia from April 1, 2023, to July 1, 2023. The study was approved by the Biomedical Ethical Committee, Umm Al-Qura University, Saudi Arabia (approval number: HAPO-02-K-012-2023-05-1610). Convenience sampling was done by distributing an online self-designed questionnaire developed and modiﬁed based on previously published studies [2,14,15]. The invited participants included all clinical years’ training students, interns, trainees (Saudi board residents and dental assistant program trainees), and dental assistants in the hospital, who were counted as 265. Trainees in our hospital were mostly in the age range of 20-32 years and hence, most of our study respondents were of this age. To ensure maximal participation, the questionnaire link was sent by email to all study participants.

Sample size determination

A sample size of ≥ 157 was calculated based on the total population of 265 trainees at the Dental Teaching Hospital, College of Dental Medicine, Umm Al-Qura University. The sample size calculation was done at a 95% confidence level and a confidence interval of 5 [16].

Study questionnaire

The questionnaire was prepared in Google Forms (Google LLC, Mountain View, California, United States) comprising 21 items in five sections, and was distributed via email and WhatsApp (Meta Platforms, Inc., Menlo Park, California, United States). The first section included an introduction to the research aim that emphasized confidentiality and considered informed consent. The second section included three questions on demographic data (age, gender, and clinical role), while the third section consisted of 10 questions to assess the knowledge and awareness about sharps injury risk, and practices related to it (the standard methods of dealing with sharps, reporting, prevention, and post-exposure prophylaxis of such injuries). The fourth (two questions) and fifth (six questions) sections had questions about previous experience with sharps injury (if any), reporting, and the actions taken by the participant following it.

To assess the level of awareness through the third-section questions, a score of 1 was given for each correct answer and 0 for incorrect answers with the best basic knowledge score being 10. A total score with a range from 0 to 10 was then calculated. The level of awareness and knowledge was categorized as poor (0-3 points), average (4-6 points), and satisfactory (7-10 points). The reporting rates of sharps injuries in the hospital in the past 24 months (from March 1, 2021, to March 1, 2023) together with the associated practices were calculated.

Validity and reliability of the questionnaire

Evaluation of the precision of survey questions was conducted by a pilot study on 10 participants after which two questions were modified; their format was edited for more clarity based on their responses. Then, to confirm the feasibility, validity, and reliability of the questionnaire, a second pilot study was conducted on another 10 participants. Google Forms was designed to accept the submission of completely answered questionnaires only and each participant was allowed a single submission to avoid bias.

Statistical analysis

Data were collected, tabulated, and statistically analyzed using IBM SPSS Statistics for Windows, Version 20.0 (Released 2011; IBM Corp., Armonk, New York, United States). Descriptive statistics were used to describe the demographic data where frequency and percentage were determined. Awareness scores of study participants were described by the mean and the Chi-square test was used for comparison between different categorical variables. A p-value of ≤0.05 was considered signiﬁcant.

## Results

Demographic data of total participants

The total number of responders was 182 (68.7% response rate) with 76.4% (n=139) of them aged 20-26 years and 69.8% (n=127) being females. The highest percentage (20.9%, n=38) of them was Grade 4 students and the lowest percentage was Saudi board students (2.7%, n=5) (Table 1).

**Table 1:** Frequency and percentage distribution of study participants’ demographic data (n= 182)

Demographic data	Frequency (n)	Percentage (%)
Age in years		
20-26	139	76.4
27-32	34	18.7
more than 32	9	4.9
Gender		
Male	55	30.2
Female	127	69.8
Clinical role		
Grade 4 student	38	20.9
Grade 5 student	34	18.7
Grade 6 student	24	13.2
Intern	32	17.6
Dental assistant	16	8.8
Dental assistant program trainee	33	18.1
Saudi board residents	5	2.7

Demographic data of participants with a history of sharps injury

The mean awareness score was satisfactory in 117 participants (64.3%), average in 64 (35.2%), and poor in one Grade 4 male participant (0.5%) with a non-significant difference between them as regards to different age groups (X2= 0.0471, p=0.976), gender (X2= 2.738, p=0.254), or clinical role (X2= 10.906, p=0.537) (Table 2).

**Table 2:** Mean awareness score versus demographic data of total study participants

	Mean Awareness score						X^2^	P- value
	Poor		Average		Satisfactory			
	n	%	n	%	n	%		
Age in years							0.471	0.976
20-26	1	0.5	48	26.4	90	49.5		
27-32	0	0	13	7.1	21	11.5		
more than 32	0	0	3	1.6	6	3.3		
Gender							2.738	0.254
Male	1	0.5	21	11.5	33	18.1		
Female	0	0	43	23.6	84	46.2		
Clinical role							10.906	0.537
Grade 4 student	1	0.5	18	9.9	19	10.4		
Grade 5 student	0	0	12	6.6	22	12.1		
Grade 6 student	0	0	5	2.7	19	10.4		
Intern	0	0	8	4.4	24	13.2		
Dental assistant	0	0	7	3.8	9	4.9		
Dental assistant program trainee	0	0	12	6.6	21	11.5		
Saudi board residents	0	0	2	1.1	3	1.3		

**Figure 1:**
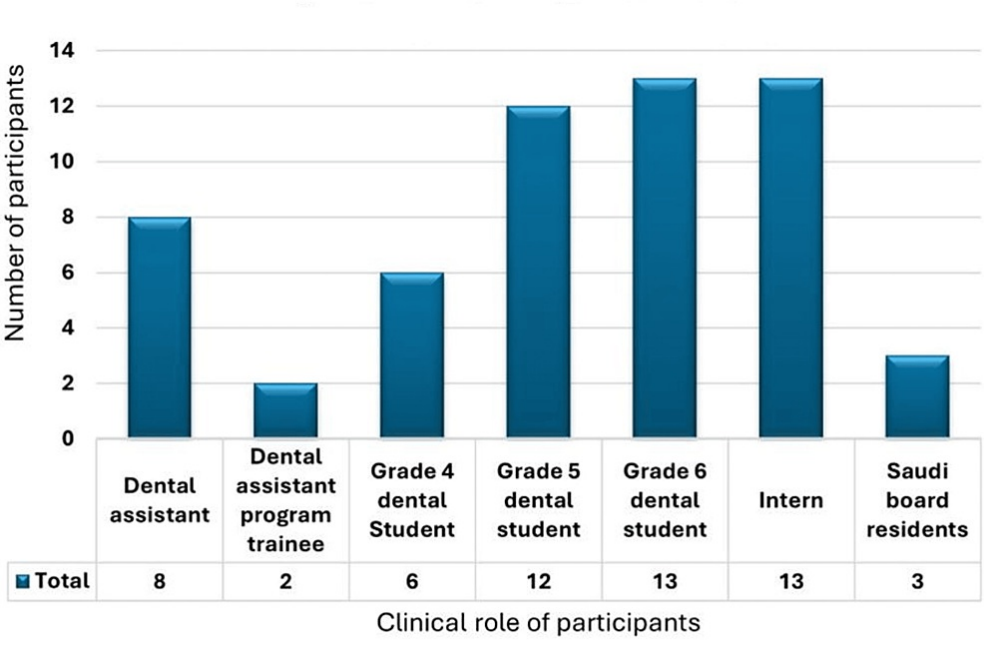
Clinical role of participants with previous exposure to sharps/needle stick injury

Out of the study participants, 57 (31.3%) had previous exposure to NSI or sharps injury while 125 (68.7%) had no history of exposure. Among the exposed participants, 52.6% (n=30) were injured in the past 12 months and the sharp item was contaminated in 66.7% (n=38) of them. The injury was superficial in 78.9% (n=45); 33.3% (n=19) were injured during injecting anesthesia while 1.8% (n=1) were injured during need disposal, surgical extraction, or surgical suture (Table 3). Interns and sixth-year students constituted the most injured participants while the dental assistant program trainees were the least injured (Figure 1). Comparison between the mean awareness score of participants with a history of sharps injury and those with no history of exposure revealed non-significant differences between both groups (X2= 2.277, p=0.320).

**Table 3:** Frequency and percentage distribution of exposed study participants’ demographic data (n= 57)

Demographic data	Frequency (n)	Percentage (%)
When did you get a sharps injury?		
Between March 2021 and March 2022	30	52.6
Between March 2022 and March 2023	27	47.4
Was the sharp item causing the injury contaminated (used in the patient treatment)?		
No	19	33.3
Yes	38	66.7
What was the type of this injury?		
Superficial	45	78.9
Moderate	7	12.3
Severe	5	8.8
What was the clinical procedure involved with your previous sharps injury exposure?		
Chairside assistance	7	12.3
Endodontic treatment	4	7.0
Injecting local anesthesia	19	33.3
Needle disposal	1	1.8
Prosthodontic treatment	4	7.0
Restorative treatment	11	19.3
Surgical extraction	1	1.8
Surgical suture	1	1.8
Periodontal treatment	2	3.5
Other	7	12.3

Comparison of knowledge between exposed and non-exposed participants

Among the study participants, non-significant differences were detected between those who were exposed to NSI/sharps injury and those who were non-exposed with regard to proper knowledge, except for the safe technique of recapping needle, in which the majority (96%, n=120) of non-exposed participants had proper knowledge in comparison to the exposed group (87.7%, n=50) with a significant difference (X2= 4.359, p=0.037) (Table 4).

**Table 4:** Comparison between exposed and non-exposed participants with regard to proper knowledge * Significance difference p <0.05

Knowledge question	Percentage of participants having proper knowledge			X^2^	P-value
	Total participants (n= 182)	Participants with needle stick injury (n=57)	Participants with no needle stick injury (n=125)		
Which diseases are transmitted by sharps injury?	165 (90.7%)	52 (91.2%)	113 (90.4%)	0.032	0.859
Which virus carries the highest risk of transmission by sharps injury?	105 (57.7%)	34 (59.6%)	71 (56.8%)	0.130	0.718
Should the needle be recapped/bent after use?	150 (82.4%)	47 (82.5%)	103 (82.4%)	0.001	0.993
In the case of recapping needles, which of the following techniques is safe? ((Proper knowledge (one hand))	170 (93.4%)	50 (87.7%)	120 (96%)	4.359	0.037*
Should needles be discarded immediately after use?	173 (95.1%)	52 (91.2%)	121 (96.8%)	2.586	0.108
What type of measures must be taken after a needle stick injury?	136 (74.7%)	44 (77.2%)	92 (73.6%)	0.268	0.605
Do you think it is necessary to report sharps injury incidents to the infection control team?	179 (98.4%)	56 (98.2%)	123 (98.4%)	0.006	0.940
Does wearing gloves reduce the incidence of sharps injury?	108 (59.3%)	32 (56.1%)	76 (60.8%)	0.352	0.553
Do you think that post-exposure prophylaxis is necessary?	174 (95.6%)	53 (93%)	121 (96.8%)	1.358	0.244

**Figure 2:**
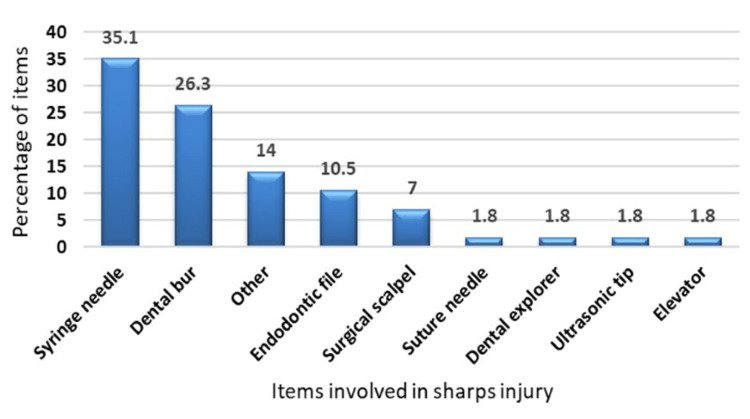
The percentage distribution of items involved with sharps injury

**Figure 3:**
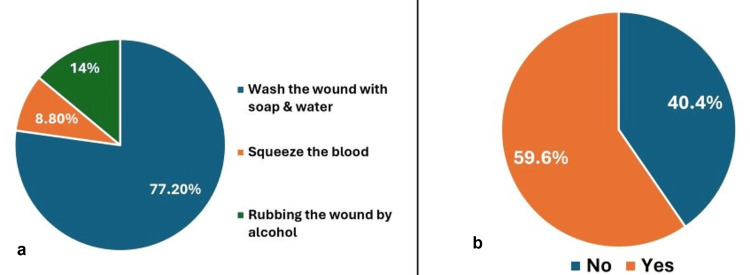
(a) Percentage distribution of actions taken after sharps injury; (b) Percentage reporting sharps injury

Among participants with a history of exposure to sharps injury, the most common item causing injury was a syringe needle (35.1%, n=20) while the items causing the least injuries were dental explorer (1.8%, n=1), suture needle (1.8%, n=1), elevator (1.8%, n=1), and ultrasonic tip (1.8%, n=1) (Figure 2). After an injury, 77.2% (n=44) washed their hands with soap and water (Figure 3a) and only 59.6% (n=34) reported the injury to the infection control unit (Figure 3b).

Comparison between mean awareness score and action taken after exposure and reporting

A significant difference was observed among various levels of mean awareness score of participants exposed to injury and action taken just after the injury (X2= 15.369. p=0.004). While non-significant difference (X2= 2.285. p=0.319) was detected between them with regard to reporting to the infection control unit (Table 5).

**Table 5:** Mean awareness score of participants with history of sharps injury with regard to action taken after exposure and reporting * Significance difference p <0.05

	Mean Awareness score						X^2^	P-value
	Poor		Average		Satisfactory			
	n	%	n	%	n	%		
What was the action taken just after the injury?								
Washing the wound with soap & water	0	0	11	19.3	33	57.9	15.369	0.004*
Squeezing the blood	0	0	2	3.5	3	5.3		
Rubbing the wound with alcohol	1	1.8	6	10.5	1	1.8		
Did you report the injury to the infection control unit?								
No	0	0	10	17.5	13	22.8	2.285	0.319
Yes	1	1.8	9	15.8	24	42.1		

## Discussion

A dental career is one of the most desirable professions nowadays. However, many unwanted occupational health hazards still exist and continue to persist in dental practice. The most important hazard is exposure to infections via percutaneous sharps and NSIs. These injuries could be attributed to the routine use of sharp instruments, that are always contaminated by blood and saliva, in dental treatment. Another important cause of exposure is wrong recapping practice after use as well as handling sharps while cleaning or unsafe disposal of the needle in a safety box [5]. It is crucial for all dental colleges to have accurate data on accidental injuries during training to help their dental healthcare practitioners realize the potential occupational hazards and their proper management and to improve the quality of dental care [17].

The present study was conducted on 182 dental students, interns, trainees, and assistants with a response rate of 68.7% of the total sample. About half (52.7%, n=96) of the participants were clinical-year students in different grades. The highest percentages of them were females aged 20-26 years. The response rate in the current study was closely similar to the majority of the previous studies that used self-administered questionnaires for data collection, where the rate was around 70% [4].

The mean awareness score in the current study was satisfactory in more than 60% (n=117) of the participants and average in about 35% (n=64). This is at par with the study by Othieno et al. (57.8%), which included 80 dental students in Uganda [8], and better than that recorded in a study in India in which knowledge about NSI risks and the use of preventive measures were found to be inadequate [18]. However, study participants in both studies were confined to dental students only.

Studies extended to include other groups, either dental students and interns, in India [19] or dental practitioners, students, and auxiliary staff in Pakistan [20] concluded that the knowledge of their study participants regarding NSI risks and the use of preventive measures was inadequate. The acceptable good awareness scores in the present study could be explained by the efforts driven by the infection control unit at our hospital for the annual training of students and all categories of dental practitioners on infection control guidelines before clinical practice.

On the other hand, one of the most important observations in the current study is the poor awareness detected in only one student. The student who had poor knowledge was a fourth-year student, and this could be attributed to the fact the first three years in our faculty are academic and the fourth year is the first professional year in our curriculum.

Regarding exposure to sharps injury, 31.3% (n=57) of total participants in the current study had previous exposure in the last two years. This percentage is similar to that reported in an earlier Indian study of dental students (35%) [20] but much lower than that reported in a study of dental students and interns in Pakistan (63%) [21] and higher than that found in a study of dental students in Qassim (27.6%) [13]. Differences between studies with regard to the prevalence of sharps injury could be attributed to different categories of dental practitioners included in each study, the period during which the prevalence is calculated, and infection control policy and training programs provided to the participants in each study.

The most common cause of injury in the present study was NSI while injecting anesthesia (35.1%, n=20), which is in tune with a previous study on dental students in China [2]. Similarly, a 19-year Japanese report on dental care providers concluded that syringe needles were the most frequent cause of injury among them [22]. The increased risk of NSIs was previously explained by the fact that most dental procedures are performed under local anesthesia with multiple injections over the course of treatment either during tooth preparation, endodontic treatment, or oral surgery. Lack of experience during aspirating syringes could lead to insufficient anesthesia and sudden jerky movement of the patient, which could predispose to such injuries [4]. Another key factor of high NSI incidence during dental practice is the use of non-disposable metallic syringes; where the practitioner must re-sheath the needle to dismantle the syringe and allow its parts to be autoclaved [23].To prevent NSIs, metallic syringes should be substituted by safety or self-sapping syringes. Although the majority of the items causing injuries in the present study were contaminated, fortunately, the majority were superficial.

Interns and sixth-year students were the most injured participants in our study. The increased incidence among interns supports the previous explanation that the inexperience of dental interns, besides the required number of cases for evaluating their performance increases their stress during work, especially in the absence of chairside assistance [4,24]. Another contributing factor to increased exposure in dental interns is a lapse in concentration where they are less focused when practicing [25]. Regarding students, this could be explained by the previous suggestions that insufﬁcient manual experience to deal with the instruments together with the enthusiasm to perform invasive techniques might increase the incidence of injury [4].

In the current study, a non-significant difference was detected with regard to proper knowledge between participants with a history of exposure and those without a history of exposure to sharps injury except for the safe technique of recapping needles. This confirms that all practitioners in our hospital have the same level of awareness.

As recapping needles is accountable for the largest proportion of NSIs, the data were analyzed in the present study, which showed that 93.4% (n=170) of the total participants correctly answered the safe needle recapping technique. This percentage was slightly higher than that reported in the study in Qassim (83%) [13]. Further analysis of the clinical role of 12 participants who wrongly answered in our study revealed that they comprised four dental assistant program trainees, four dental assistants, two fourth-grade students, one intern, and one Saudi board resident). This observation raises the need for individual counseling sessions for such practitioners to modify their bad practices as well as the need to pay more attention to such potentially risky behavior in future training in our hospital.

Among participants with a history of sharps injury, the most common item causing injury in the current study was a syringe needle. This is similar to a study on dental students in a South African university [26]. The second most frequent item causing injury in the present study was the dental bur, which supports a previous suggestion that bur punctures were another common cause of sharps injury while dental practitioners pick up an instrument from the bracket table. So, adjustments of the bracket table and positioning of hand-piece holders should be modified and dental practitioners should remove the burs in a timely manner [27].

Reporting NSIs is important to rapidly evaluate the required post-exposure prophylaxis for the injured person and reduce the accompanied anxiety. It also allows practitioners to recognize hazardous devices or procedures and work in a manner that reduces the risk of future exposure. So, according to the infection control policy in our hospital, all practitioners are trained during the annual training sessions to report the injury to the infection control supervisor and fill in the form available in the hospital. Although 98.2% (n=56) of injured participants knew that it is necessary to report sharps injury incidents to the infection control team and 93% (n=53) of them knew that post-exposure prophylaxis is necessary, only 59.6% (n=34) reported injury. This reporting rate is similar to that recorded in a New Zealand study, where 67.2% of injured students reported their injury [28], and much lower than that in a South African study (92%) [26]. The reporting rate in the present study could be attributed to underestimation of the condition as more than 76% (n=45) of them had superficial injuries.

The under-reporting of NSIs or non-reporting has a high prevalence all over the world. In a Nigerian study, no injured students formally reported the exposure [29], and in a Chinese study, only one dental student out of the injured study participants reported the injury [27]. Literature cited that reasons for under-reporting could be due to fear of stigmatization, ignorance of participants that all injuries must be reported, bad practice, or low perception of the associated risk [30].

After an injury, 77.2% (n=44) of our study participants cited that they washed their hands with soap and water with a significantly higher percentage of them having a satisfactory mean awareness score. Similarly, 85.2% of students in Iraq washed their wounds with soap and water [10]. On the other hand, an Indian study of dental students found that only 12% of injured students washed the site injury with soap and water immediately after the injury [31].

The present study has some limitations; first, the sample size included only those belonging to one dental teaching hospital, so the results could not be generalized. Second, a questionnaire provides self-reported data that could be subject to recall bias associated with a low strength of evidence. Third, it did not explore the circumstances of injuries or the causes of non-reporting among participants. However, despite these limitations, it is one of the few studies to involve dental students and trainees together with dental assistants in the identiﬁcation of procedures and instruments associated with sharps injury.

## Conclusions

A high percentage of clinical-year dental students, interns, trainees, and assistants have satisfactory awareness, and a lesser percentage has average awareness about the risk of sharps injury and the preventive measures with high under-reporting rates. We thus recommend the enforcement of pre-practice education and training to increase awareness among all categories of trainees about potentially risky behavior, especially two-handed needle recapping and reporting protocol. Together with revisiting the faculty teaching curriculum to include infection control preclinical courses to ensure safe practice and improve the quality of dental care.
